# Evaluating the Adequacy of Gravity Models as a Description of Human Mobility for Epidemic Modelling

**DOI:** 10.1371/journal.pcbi.1002699

**Published:** 2012-10-18

**Authors:** James Truscott, Neil M. Ferguson

**Affiliations:** MRC Centre for Outbreak Analysis and Modelling, Imperial College London, London, United Kingdom; University of Michigan and Howard Hughes Med. Inst., United States of America

## Abstract

Gravity models have a long history of use in describing and forecasting the movements of people as well as goods and services, making them a natural basis for disease transmission rates over distance. In agent-based micro-simulations, gravity models can be directly used to represent movement of individuals and hence disease. In this paper, we consider a range of gravity models as fits to movement data from the UK and the US. We examine the ability of synthetic networks generated from fitted models to match those from the data in terms of epidemic behaviour; in particular, times to first infection. For both datasets, best fits are obtained with a two-piece ‘matched’ power law distance distribution. Epidemics on synthetic UK networks match well those on data networks across all but the smallest nodes for a range of aggregation levels. We derive an expression for time to infection between nodes in terms of epidemiological and network parameters which illuminates the influence of network clustering in spread across networks and suggests an approximate relationship between the log-likelihood deviance of model fit and the match times to infection between synthetic and data networks. On synthetic US networks, the match in epidemic behaviour is initially poor and sensitive to the initially infected node. Analysis of times to infection indicates a failure of models to capture infrequent long-range contact between large nodes. An assortative model based on node population size captures this heterogeneity, considerably improving the epidemiological match between synthetic and data networks.

## Author Summary

An accurate representation of disease transmission between spatially-distinct regions is an essential part of modelling epidemic behaviour on a national or international scale. Gravity models, which describe movement fluxes between regions in terms of their populations and distance from each other, have a history of successful use in the geography and economics and are increasingly used in epidemiology. We look at the ability of a range of gravity models to fit human movement data from the UK and the US. In particular, we compare the behaviour of a simple flu-like epidemic model on synthetic networks generated by fitted gravity models and on the original network present in the data, using time to first infection. For UK data, epidemic behaviour on synthetic networks matches that on the original data quite closely. For US movement data, synthetic networks perform much worse. We develop an analytic expression for infection time between two regions which indicates that our gravity models fail to capture long range connections between large populations. A model with assortative mixing based on population size greatly improves the match between synthetic and data networks.

## Introduction

Gravity models of population movement characterize the distribution of trips between discrete locations, based on the populations of the origin and destination and the distance between them. The use of gravity models to describe movement between population centres can be traced back at least as far as the work of Zipf in the 1940's [1]. In this work, Zipf provides a theoretical motivation for movement between cities 1 and 2 being governed by a *P_1_P_2_/d* relationship, where *P* is the respective city population and *d* is separation distance. He also identifies this relationship in passenger and freight movements between US cities [1]. Until recently, the main areas of application for gravity models has been in analyzing and forecasting the demand for goods and services in spatially distributed populations [2]. Consequently, much of the theoretical work has focused on the modeling of journey costs and discrete choice models [3].

More recently, gravity models have been adapted to describe the spread of a range of biological agents, such as invasive species [4] and plant pathogens [5]. Of particular interest in the context of this paper is their use as a description of infectious disease transmission between regions. As a description of movement between spatially distinct populations, gravity models offer a simple model of disease transmission strength between meta-populations. Xia et al. model the dynamics of pre-vaccination measles as city meta populations connected by a gravity-based movement of infectious individuals [6]. The model succeeds in capturing most of the spatiotemporal properties of epidemics, including case rates, periodicity and fade-out behaviour. A similarly structured model was applied to seasonal influenza data in the US by Viboud et al. [7]. In this case, it was found that fitting the underlying gravity model to commuting data successfully captured the observed synchrony in epidemics as a function of distance, population size and transmission. Gravity models are now increasingly used in both metapopulation- and individual-based epidemic micro-simulations [8]–[11].

The aim of this paper is to look at the ability of gravity models to represent commuter movement data in the UK and US to the fidelity needed to capture expected patterns of epidemic spread. We examine which models best capture the statistical properties of the data and what aspects are poorly represented. We address how the choice of level of spatial aggregation influences the fit of models. Most importantly, we examine the behaviour of a simple SIR epidemic on synthetic networks reconstructed from fitted gravity models and ask how epidemic behaviour depends on the underlying model and on the level of aggregation.

## Materials and Methods

### UK commuting data

The UK population and commuting data is taken from the 1991 census. The data set combines information for England and Wales collected by the Office for National Statistics and Scottish data from the General Register Office for Scotland [12], [13]. The smallest region of aggregation of data available is the Census Area Statistics Ward, corresponding to the electoral wards which define the basic political and administrative geographical units in the UK. We look at the fit of models at three different levels of aggregation, featuring wards, district and counties as the basic regions. We concentrate on the district level, as districts correspond well with individual cities and are comparable in many ways to counties in the US data-set.

Information on commuting patterns comes from the associated Special Workplace Statistics for both Scotland and England and Wales. The data sets comprise the responses from a randomly chosen 10% of the surveyed population who were asked for the location of their place of work and their means of transport for commuting. From this can be constructed the flow of commuters between any two pairs of wards (in this paper we aggregate across modes of transport to recover the total flow).

The models we fit to the data are functions of the destination work population, so we exclude the small fraction of wards (1%) that have no commuter population. For the remaining wards, our data set consists of a population of wards with resident population and location (as northing and easting) and a set of commuting sub-populations between pairs of wards. This data set can easily be spatially aggregated to the district of county level.

We have chosen to use the 1991 census data as opposed to the more recent 2001 set, which entails two potential drawbacks. Firstly the data is a decade older, but more importantly only 10% of commuting data was obtained in 1991. Use of the 2001 data is problematical, however, due to disclosure control methods introduced in that year. The so-called ‘small cell adjustment method’ (SCAM) involves the adjustment of small cell counts (1 or 2) in the data to either 0 or 3 (precise details of the algorithm have not been released). Stillwell and Duke-Williams discuss some of the consequences arising from SCAM [14]. Among those relevant to the current work are that small commuter counts are more likely at longer distances and these are particularly important for fitting the power-law distributions used in our models and also that small numbers of individuals may still trigger disproportionately large local epidemics. A further issue is that SCAM is not applied to movements with origins in Scotland and is hence spatially inhomogeneous.

### US commuting data

US population and commuting data is taken from the US Census in 2000. The data is available from the US Census Bureau web-site [15]. With regard to working habits, the census asks participants to identify the location of the place they worked at most frequently in the previous week. Hence we might expect the resulting data to contain more irregular and long-distance journeys than the UK data. Commuting data for 2000 at the county level was retrieved from the site along with populations and geographical centroid location for each county. We consider only counties from the 48 contiguous states and exclude all movements to US territories. The mean population of the included counties is approximately 90,000 residents.

### Movement models

We look at movement models that calculate the probability, *p_ij_*, of a journey from node *i* to node *j*

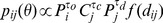
(1)where *P_i_* is the resident population, *C_i_* is the population who work in node *i* (regardless of where they live) and *θ* is a vector of parameters. The function *f(d)* is a distance kernel which encapsulates the effect of separation on the probability of a journey between two locations. We normalize to impose the constraint 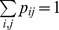.

As well as this ‘unconstrained’ model, we consider a ‘constrained’ model which assumes that the probability of a journey emanating from node *i* (including those within node *i*), *q_i_*, is matched to that in the data. That is,
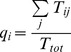
where *T_ij_* is the flow from node *i* to *j* and *T_tot_* is the total number of journeys in the data. We then express the probability of a particular journey in a conditional form, 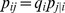, where
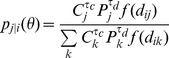
(2)By setting parameters to zero, we can examine a range of sub-models. We look at models of distance interaction of two forms, smooth kernel (SK),
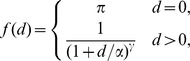
and a two-piece ‘matched’ kernel (MK),
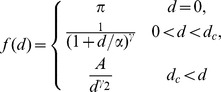
where *π* is a point probability mass for commuting within the node of residence (*d* = 0), *α* is a distance scale below which the kernel function saturates, and *γ*, *γ_2_* are power parameters. Here *A* is a function of the other parameters such that *f*(*d*) is continuous at *d_c_*.

These models are fitted to the movement data by constructing a likelihood function for the data and maximizing using standard MCMC techniques. We use a multinomial likelihood for the distribution of journeys among nodes
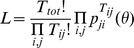
Discarding terms independent of the movement model gives a log-likelihood of
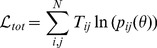
(3)In the case of the locally-constrained model, the log-likelihood can be split into two terms
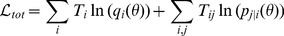
(4)If the set of *q_i_* are considered as *N* additional independent parameters, it is easy to show that local constraints correspond to the maximization of the likelihood with respect to *q_i_*, allowing a direct comparison of the two model types.

The raw commuting data can be represented as a weighted graph, the nodes of which are the locations represented in the data. Edge weight is then just the number of reported journeys between two nodes. One can then simulate an epidemic occurring on this data-derived network. An equivalent interpretation is that of a metapopulation, where the patches are the locations in the data and the origin-destination flow matrix is used to construct the patch-to-patch coupling matrix. Similarly, we can construct synthetic networks from the fitted movement models and compare the dynamics of epidemics on those networks with the dynamics of epidemics on the data-derived network.

### Epidemic model

In order to make these comparisons, we employ a simple stochastic SIR (susceptible-infected-recovered) model such as might be used to describe the spread of influenza. A connection from node *i* to node *j* consists of a population, *N_ij_*, resident in *i* and working in *j*. To simplify the model, we assume that all residents of a node are working (in reality, approximately 40% of the resident population travels to work). Of this population, *S_ij_*(*t*) and *I_ij_*(*t*) are the number of individuals who are susceptible and infected at time *t*, respectively. The susceptible population is subject to forces of infection from the node within which they reside, 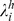, and the one at which they work, 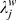. Both the working and resident populations are assumed to be well mixed and hence the forces of infection are given by
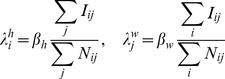
Infected individuals recover into a removed class at a rate σ. Hence in a naïve population, R_0_ = (*β_h_+β_w_*)/*σ*, neglecting local saturation effects. Unless otherwise stated, we use the following parameter values: *β_h_* = 0.5/d, *β_w_* = 0.4/d and *σ* = 0.5/d, giving a generation time of 2 days and an R_0_ of 1.8.

Comparison of the behaviour of the epidemic model on different networks is based on the times to first infection for network nodes from a given initial infection site. Under certain simplifying assumptions, an approximation for the mean time to infection between two nodes can be calculated for the above epidemiological model.
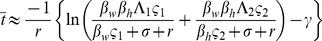
(5)The parameters *Λ_1,2_* and ς*_1,2_* represent network properties, such as the fraction of journeys between the two nodes. r = *β_h_*+*β_w_*+*σ* is the epidemic growth rate in a large node. The equation illustrates how epidemiological effects combine with network properties to determine the speed of infection across a connection. The details of the analysis and the definitions of the parameters can be found in the Supplementary Information Text S1.

## Results

### UK fits

Table 1 shows the maximum likelihood values and associated parameter estimators for a range of models at the district level of aggregation in the UK. The biggest influence on log-likelihood is whether local constraints are imposed. For a given kernel type, a locally constrained model has a greater log-likelihood by a margin of approximately 230,000. The locally constrained model effectively has an extra parameter for each node (the number of workers living there). Model comparison statistics (such as the Akaike information Criterion) offset the number of model parameters against the log likelihood, but these 456 additional degrees of freedom are clearly insufficient to account for the increase in likelihood. We have omitted further discussion of models which include an exponent on the destination population, *τ_d_*. The improvement in likelihood over a model with only commuter population dependence is minimal (∼2500) and not discernible in the statistics of flow distributions or behaviour of the epidemiological model.

**Table 1:** Maximum likelihood parameter estimates for different models to UK commuting data at the district level of aggregation and for the MK, locally constrained model across different levels of aggregation.

Models at district level aggregation								
Model	τ_o_	τ_c_	γ	α (km)	π	γ_2_	*d*_c_ (km)	Δ* 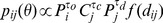 *
MK, constrained	0	0.95	6.6	35	1.3	1.53	87	−267138
SK, constrained	0	0.95	3.9	13.5	0.79	-	-	−283505
MK, unconstrained	0.23	0.72	17.4	158	4.1	1.79	70	−496648
SK, unconstrained	0.23	0.71	4.1	22	2.65	-	-	−516385
Best fit model at different aggregation levels								
County	0	0.89	19	304	2.4	1.24	130	−86286
District	0	0.95	6.6	35	1.3	1.53	87	−267138
Ward	0	0.98	4.1	8.5	1.13	1.84	87	−1499291

Log likelihood values quoted relative to that of the saturated model. Saturated model log likelihood = −9087628 (county), −14941875 (district), −24483851 (ward).

A smaller difference arises between models with a smooth offset power law kernel (SK) and those with a matched two-section kernel (MK - see the Models section). For a given constraint type, use the MK model improves maximum likelihood by approximately 20,000 for the addition of two new parameters; a critical distance, *d*_c_, beyond which an outer power, *γ_2_*, is used. In general, credible intervals for parameters are typically less than 0.5% of their maximum likelihood estimates. Intervals for π and *γ_2_* are slightly wider at around 1.5%, reflecting the smaller amount of data to estimate them Such narrow intervals reflect the large amount of data used in the fitting, rather than sensitivity in any of the observable statistics of the fitted model.

Across different levels of aggregation, the MK, locally constrained model consistently gives the best fit. Parameter values generally change monotonically as the aggregation unit is made smaller. In particular, note the trend in commuter population power, *τ_c_*, and the point mass, π, which both approach 1 as the size of the aggregation unit approaches 1, a feature we address in the discussion. The wide range of powers and scaling parameters for the inner kernel can be understood in the context of the limiting behaviour of the offset power law. For large γ, the offset power function converges to an exponential distribution.
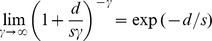
where *s* = *α/γ*. Given the magnitude of the estimates of *γ* for the MK model, the ratio s is perhaps a better estimate than *α* of the spatial scale of the inner kernel. Its size is comparable to that of a typical node at each level of aggregation.

**Figure 1:**
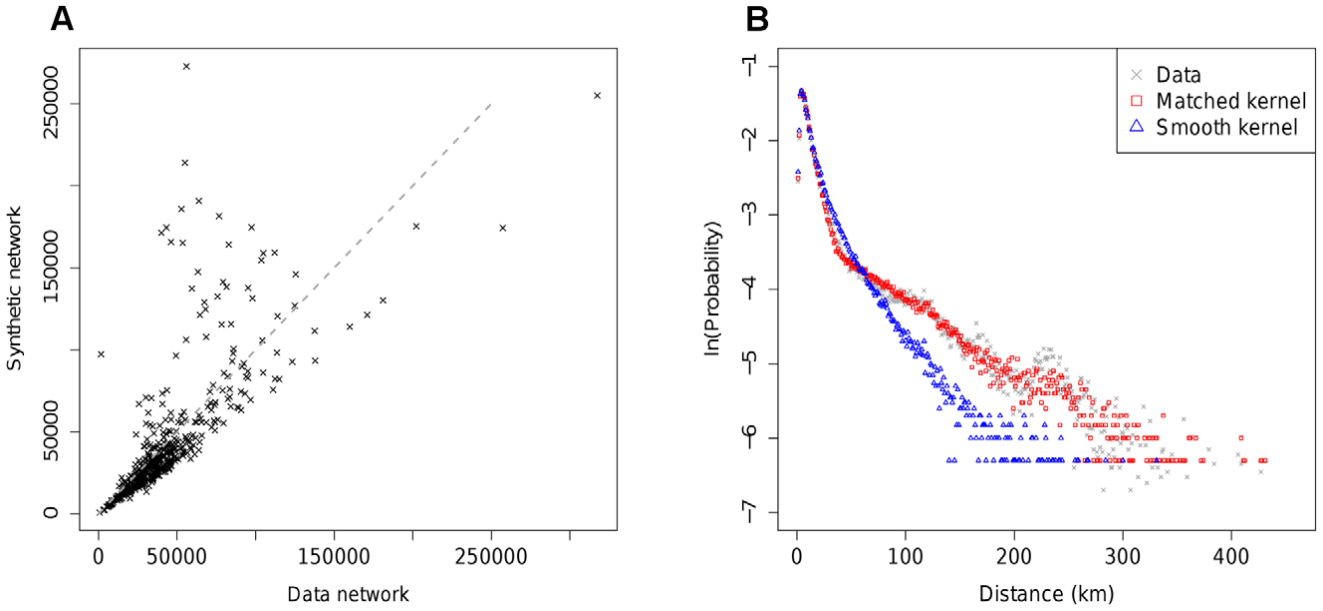
A) Comparison of observed node outflows with those generated by the globally-constrained MK model. B) Distance distribution of synthetic connections generated by SK and MK locally constrained models.

Figure 1 illustrates the source of the differences in likelihoods. From the point of view of the two part likelihood expressed in equation 4, the globally-constrained model needs to fit the total outflow from each node as well as the relative probabilities of journeys starting from each node. As shown in Figure 1A, the model generally underestimates the number of travelling workers in a node.

Figure 1B shows the distance distribution among connections in the SK and MK constrained models in comparison to that found in the data. The distribution from the movement data has a clear ‘kink’ at approximately 150 km, beyond which journeys are more common than would be expected under a pure power-law distribution. Only 1% of journeys are longer than 225 km, with 90% being less than 42 km. As a result, shorter journeys dominate the likelihood and introduce a strong bias in the longer journeys for the SK model.

**Figure 2:**
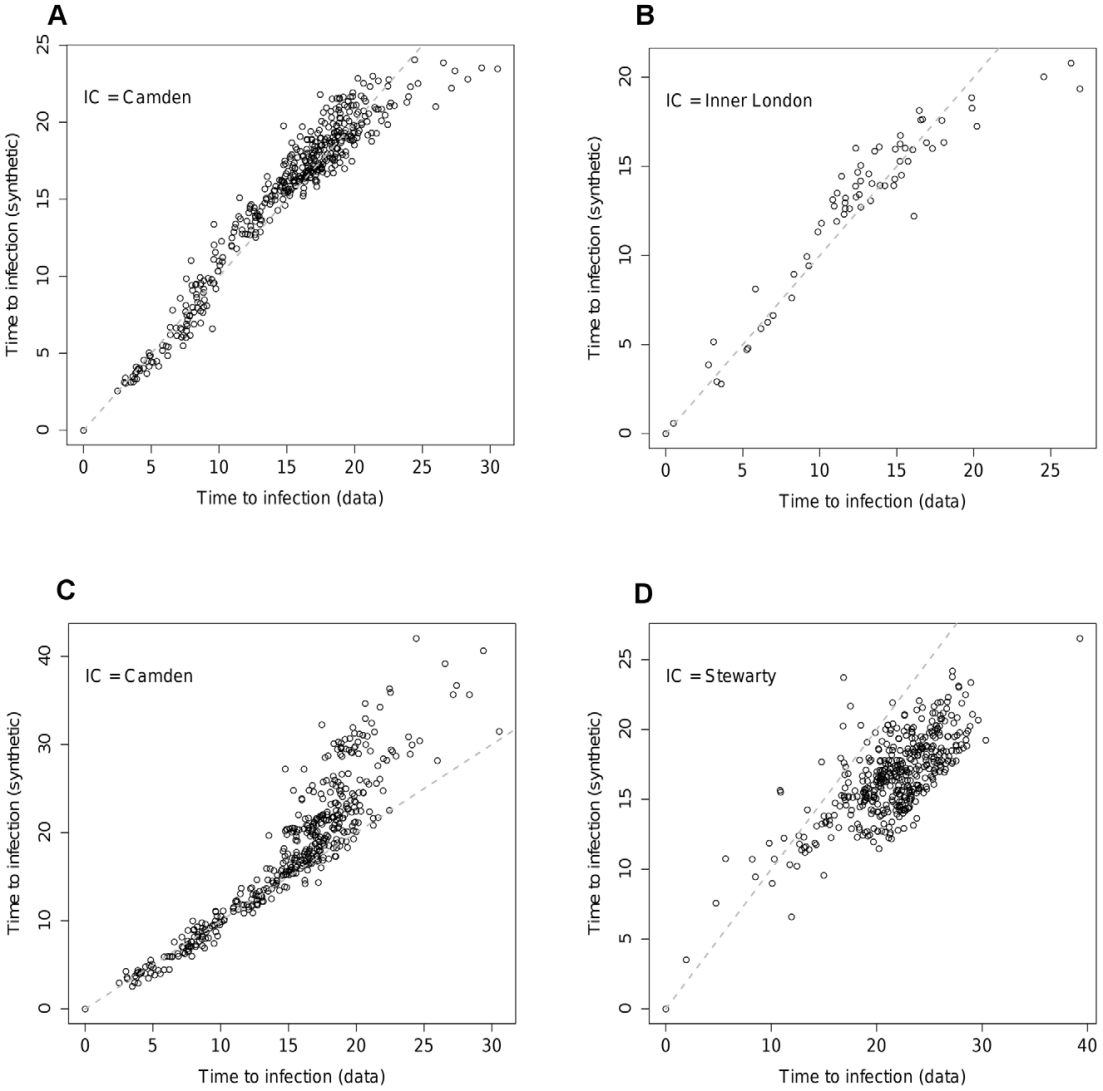
Times to infection for locally constrained MK model at A) district level and B) county level from initial seeding in Camden. C) Times to infection for smooth kernel model and data network against distance from seeding event. D) Matched kernel model times from least populous node in UK (Stewarty).

Figure 2 compares the behaviour of epidemics on synthetic networks derived from these model fits with epidemic dynamics on the network constructed using the data. Times to first infection for each node are shown, calculated as the mean time (over 100 realisations) to the first infection of a resident of the node. Figure 2A and B show the results for the MK model for epidemics started in Camden, London, at the district and county level of aggregation. The fit is quite accurate across all nodes for both aggregations, with a root mean square error of 1.6 and 1.9 days for the district and county levels respectively. In contrast, the use of the SK model has a pronounced and characteristic effect on the progress of an epidemic (Figure 2C). Times to infection match well up to approximately 15 days, at which point the infection of subsequent nodes is delayed by up to 2 weeks. This is because the SK model underestimates the degree of contact over longer distances. Figure 2C therefore indicates that the later infected nodes are infected across long distances, from some of the initially infected nodes, rather than along longer chains of short range transmissions. The good agreement of times to infection between the data and synthetic networks is generally maintained across different initial nodes. The exception is for initial nodes with very small populations. In these cases, the times to infection for other nodes is uniformly faster for the MK modelled network than the data network (see Figure 2D). The faster transmission from the smallest nodes is matched by the faster transmission to the smallest nodes (points under the line with times>20 days in Figure 2A and B) and appears to be a general feature of these models.

**Figure 3:**
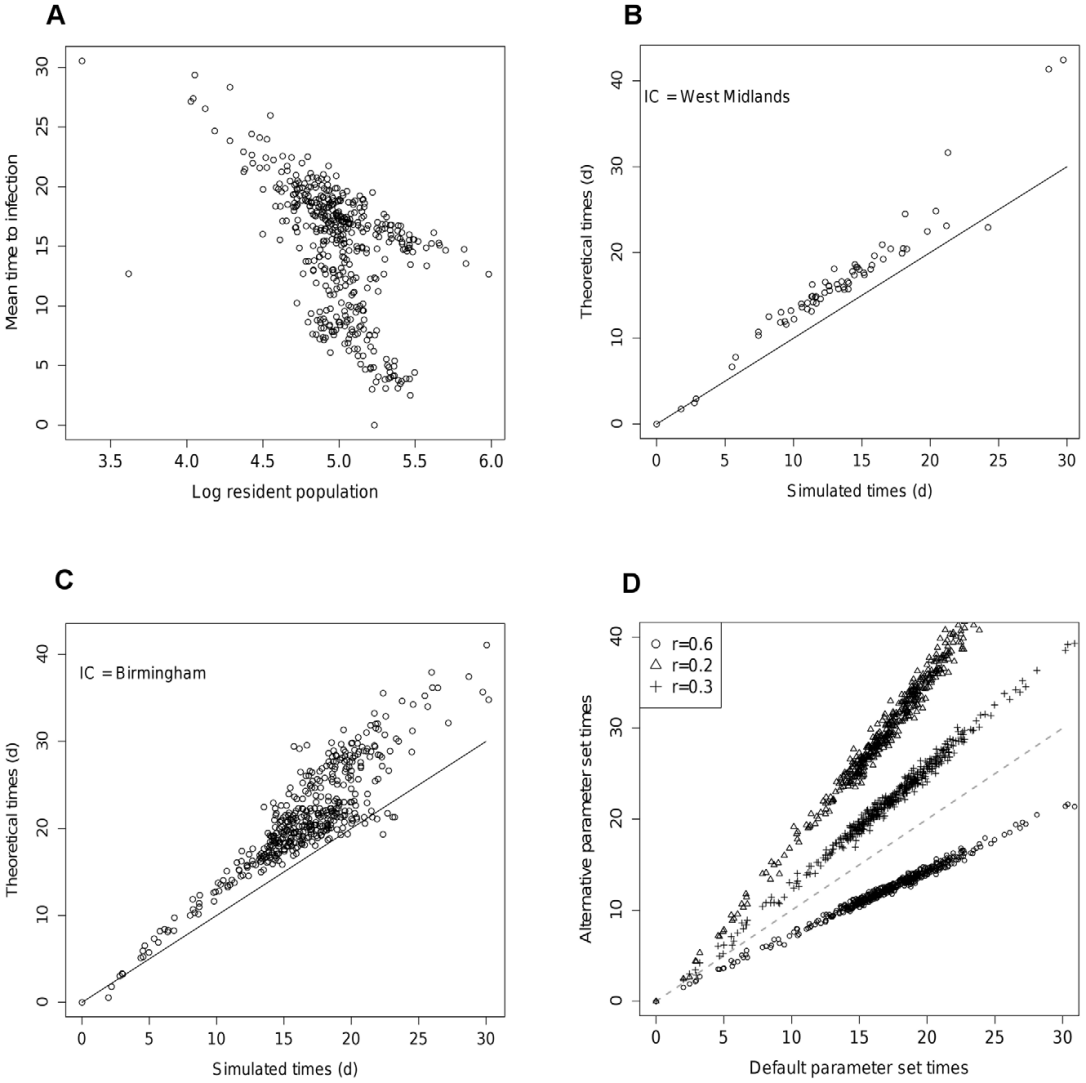
Estimates of time to infection from theory. A) Time to infection against log resident population on the data network (initial seeding: Camden). B) Theoretical against simulated times to infection across whole network at county scale as predicted by quickest path (seeding: West Midlands). C) As B) but at District level (initial seeding: Birmingham). D) Sensitivity of time to infection to value or r (default r_0_ = 0.4) initial seeding: Birmingham.

Figure 3A shows times to infection on the data network against log node population. The shape and gradient of the main diagonal band is common to all seeding points, indicating a strong linear relationship between time to infection across the network and the log population of a node. For seeding in London, a second initial band of similar gradient can be seen for times less than 10 days, representing spread from the initial seeding through the Home Counties by London commuters.

The analytical approximation for the time to infection of an epidemic process on a travel network (developed in Supplementary information text, S1) is able to shed greater light onto the influence of epidemiological processes and network structure on the behaviour of the epidemic. In general, times to infection for a particular node will depend not only on the epidemiological parameters and the movement fluxes to and from that node, but also on the structure of the travel network as a whole. Times to infection will depend on the structure in two main ways. Firstly, through how direct a route exists between two nodes and secondly on the degree of clustering. Clustering potentially allows each node to be subject to more than one force of infection from different connected nodes.

In order to investigate the importance of clustering (i.e. multiple competing sources of infection), we can construct a distance matrix between nodes with elements given by the transmission times predicted by equation 5, which implicitly assumes only a single source of infection for each node. A measure of distance between 2 points in the network is then the shortest path between those points given by the distance matrix (shortest distances can be calculated using the Floyd-Warshall algorithm [16]). Figure 3B and C compares times to infection estimated using this shortest path algorithm applied using the data network with those generated by simulating an epidemic on the same network, at the county and district level of aggregation respectively. At the county level, the agreement with theory is fairly good, suggesting that, in general, the force of infection experienced by a given node is dominated by a single infected contact and that clustering plays only a minor role. For district aggregation, agreement is good for the first 15 days, after which the theoretical predictions for many nodes are late by 7–10 days. This suggests clustering of connections is accelerating transmission, almost certainly through large conurbations. We discuss this effect in detail in the final section.

Figure 3D illustrates the sensitivity of times to infection to variation in the epidemiological parameters. As discussed in SI Text S1, theory predicts that times to infection should scale proportionally with changes in the parameter grouping 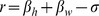. This effect can be clearly seen in Figure 3D in which times to infection for different values or *r* are plotted against those for the default value, *r_0_*. The gradient of lines in Figure 3D should be well approximated by
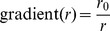
And this is the case over a range of values of *β_h_*, *β_w_* and *σ* (R^2^>90%).

### US fits

Table 2 shows the best fit parameters for a range of models to the US movement data. The order of goodness of fit is the same as that for the UK data set. Credible intervals are generally smaller than for the UK data set (<0.5% for all parameter values), reflecting the larger dataset from which they are inferred. Type of constraint is again the dominant effect with a difference in log-likelihood that cannot be accounted for by the extra effective degrees of freedom (3109 nodes in this case). There is a clear secondary effect from allowing the distance kernel to be of two sections which suggests that the distance distribution of connections may have a ‘kink’ in it similar to the UK case.

**Table 2:** Best fits for various models to US data at the county level of aggregation and the best fit for the MK model at the state level.

Model	τ_o_	τ_c_	γ	α (km)	π	γ_2_	*d*_c_ (km)	Δ* 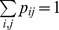 *
MK, constrained	0	0.82	20.3	293	1.19	1.8	155	−13496740
SK, constrained	0	0.81	4.17	25.7	0.39	-	-	−15797154
MK, unconstrained	0.31	0.74	5.9	146	3.78	3.4	40	−19849694
SK, unconstrained	0.32	0.73	4.5	43	1.28	-	-	−22199706
Best fit model at different aggregation levels								
US County	0	0.82	20.3	293	1.19	1.8	155	−13496740
State	0	0.59	20	322	0.4	2	35.5	−2949828

Log likelihoods are quoted relative to that of the saturated model. County-level saturated model log likelihood = −975239008, State-level saturated model log likelihood = −467836121.

**Figure 4:**
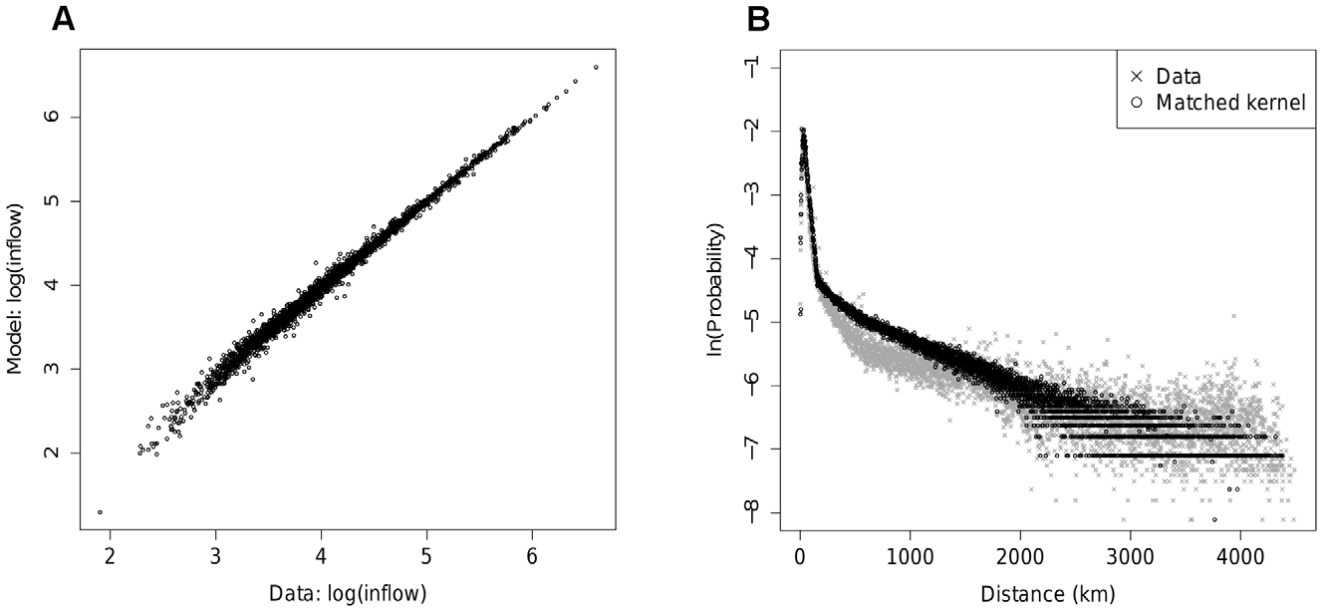
Comparison of summary statistics between the best fit MK model and data. A) Predicted inflow to nodes against actual inflow. B) Distribution of trip distances for matched kernel model and data.

Figure 4A and B illustrate the quality of synthetic networks generated from the best fitting local, matched model. There is strong agreement with data for predicted inflows to nodes (Figure 4A), but a weaker match to the distance distribution of journeys, particularly between 300 and 1200 km.

**Figure 5:**
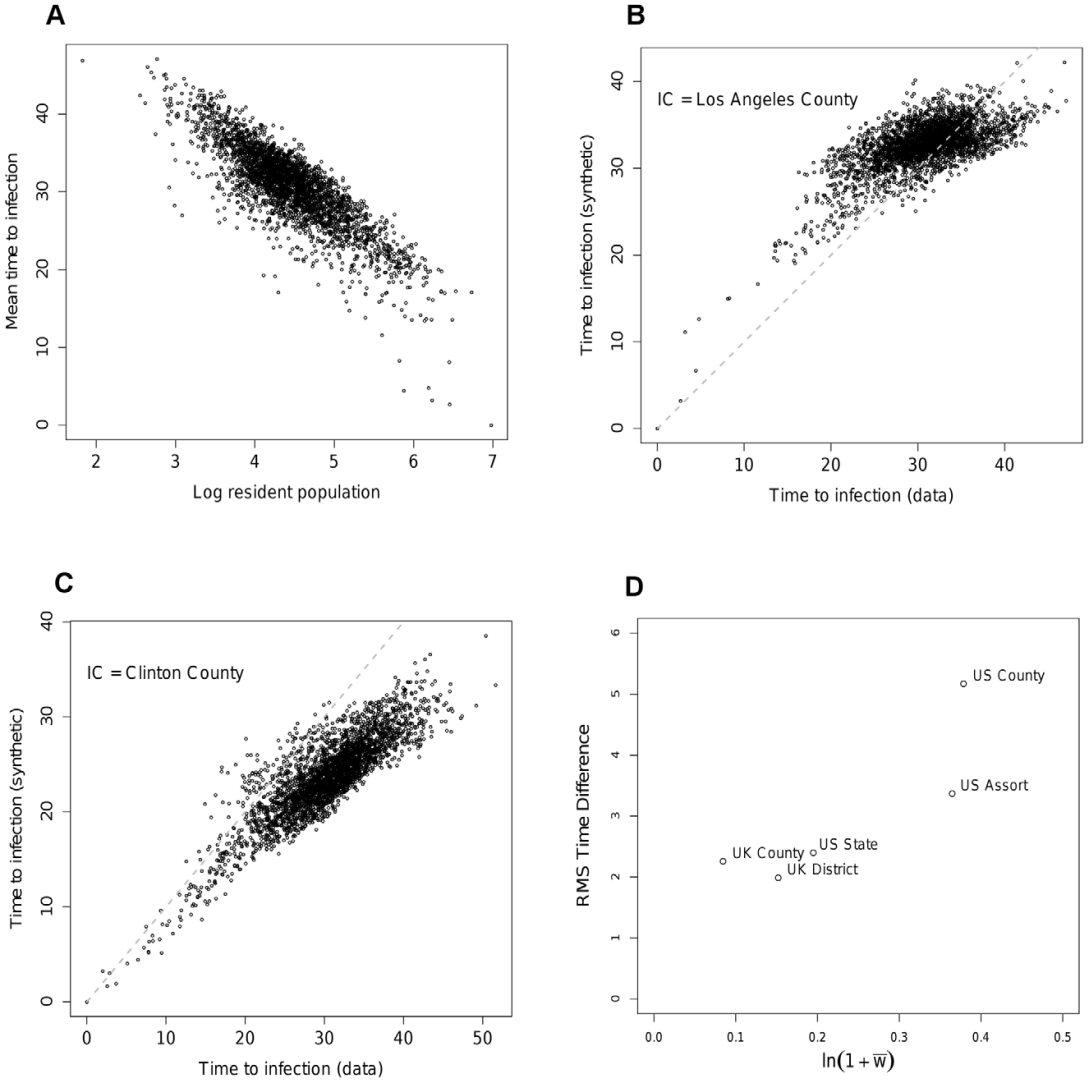
Epidemic dynamics in the US. A) Mean times to infection on the data network for counties against log population. Mean times to infection on the MK model network vs. those on the data network for epidemics initialised in B) Los Angeles County and C) Clinton County, Iowa (small dots give 95% confidence intervals on the times to infection for the data network). D) RMS difference in time to infection between data and synthetic networks (see Results section) against mean deviance for the best fit MK model on different data sets and at various aggregation levels.

Figure 5A shows mean times to infection on the data network for all counties in the continental US against the log of their populations. There is a strong linear correlation between node infection time and log population and this relationship is to a large extent independent of the initial point of infection. The effect matches that seen in the UK epidemics, but is more pronounced.

As illustrated by Figure 5B and C, the epidemic dynamics recovered from the best fit US MK model are much poorer than seen for the UK. For a significant fraction of nodes, deviations from the target behaviour are large, going beyond the 95% confidence intervals for the times to infection on the data network. The distribution of deviations is also not consistent across initial seeding points. Seeded in Los Angeles County, times to infection from the MK model are higher than for the data network for low times to infection, but too low for the nodes with longest time to infection. From Clinton County, Iowa (population approx. 50,000), infections times are uniformly too low for the synthetic network.

The significantly poorer fit in the US than in the UK is to be expected given the log likelihood values for the underlying model. In SI Text, S2, we derive an approximate measure of the goodness of fit of the mobility model which can be compared between different datasets. This mean deviance measure is based on the relative log likelihood, Δ*
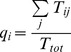
*, and is defined as
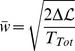
where 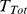 is the total number of trips in the relevant dataset. The derivation shows that the quantity 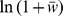 is also related to the expected deviation in time to infection across the model network. Figure 5D compares this goodness of fit measure to the corresponding measure of the goodness of fit of simulated epidemics on the synthetic network to those on the data network. This latter goodness of fit between epidemics is quantified by the root-mean-square (RMS) difference in mean times to infection between the data and synthetic networks, averaged across a range of initial nodes. Each point on the figure is based on the best fitting MK model applied to a different underlying dataset or level of spatial aggregation. Values of 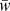 suggest that the fits of the MK model to the US data at the state level and to the UK data at the district and county level are of comparable quality. RMS differences in time to infection are also similar for these fits at 2–2.5 days. The value of 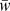 for the county level US data set is much greater, indicating a worse fit, and the RMS difference in time to infection is also much larger.

**Figure 6:**
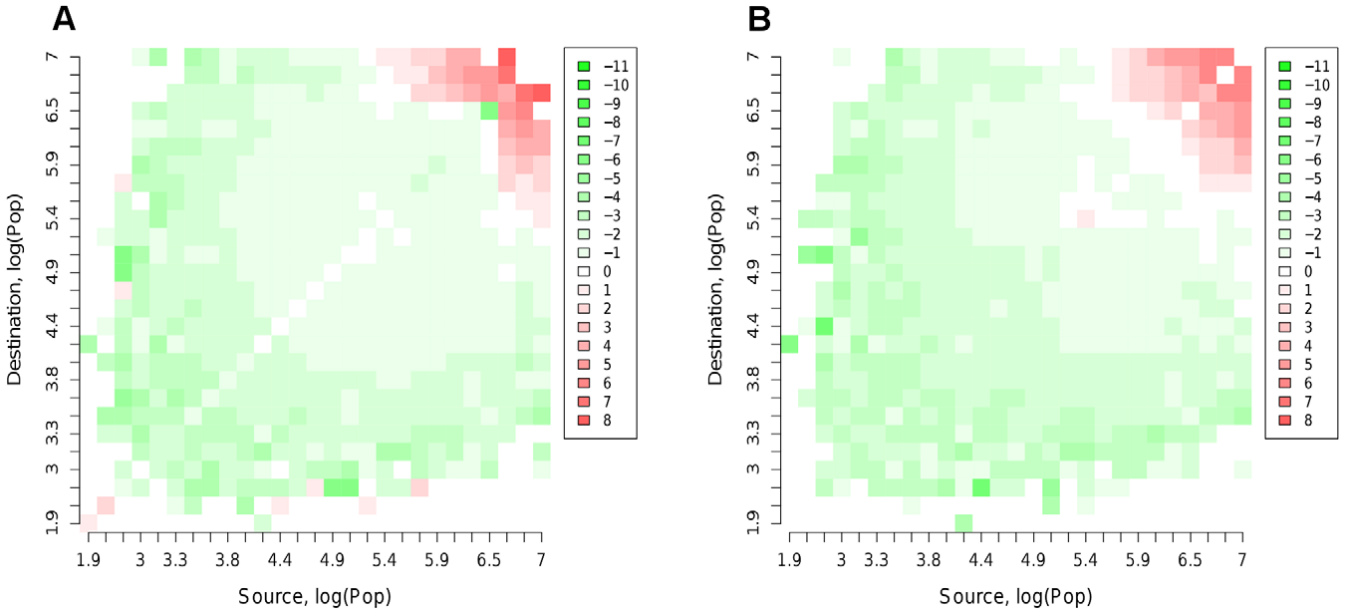
Mean time to infection difference matrix for the US. For each network, the time to infection between 2 nodes is averaged across all connections within a square. Colours in the matrix represent the difference between mean times on the A) MK network B) Assortative network and the data network. Positive values represent slower transmission on the synthetic network. Times to infection are calculated from equation 5.

There is considerable variation in the goodness of fit among epidemics started from different nodes between the MK and data US networks. This suggests that the model is failing to capture accurately some subset of the work flows in the dataset. We can use the expression for the time to infection (Equation 5) to calculate the theoretical mean time to infection for all connections in both the data network and the synthetic MK-based network to try to identify what the essential discrepancies are. The two networks differ not only in the work flows between nodes, but also in which connections are present, so it's necessary to aggregate the time to infection information to allow comparison. In Figure 6, average times to infection between pairs of nodes are shown aggregated into bins by source and destination log population size. Use of log population size is suggested both by the form of equation 5 and the strong correlation it has with infection times.

Figure 6 shows that for connections between large nodes, transmissions on the synthetic network are markedly slower than on the data network (red region). This is balanced by faster times to infection for other connections, particularly from small population source nodes. The majority of connections, represented by the centre of the diagram, fit quite well. These results suggest a distinct mechanism that is missing from the simple MK model that affects movements between highly populated nodes. Since only a small fraction of total movements is between such nodes, the effect is swamped in the likelihood. The best fitting region is for log populations between about 5.3 and 6.2, corresponding to the sources of the bulk of outflows.

To better capture the interactions between large population centres, we adapt the MK model to make it assortative with respect to node population size. The assortative model categorises nodes as large or small according to a critical size, P_c_. To allow large sparsely-distributed population centres to make contact with each other, different gravity model parameters are then fitted for large to large interactions and for all other possible contacts.

The best fit parameters for the assortative model are shown in Table 3. The main contrast with the simple MK model is in the outer power parameter, γ_2_. The previous value of 1.8 is reduced to 1.21 for movements between large nodes, but increases to above 2 for all other types of connection. This encourages longer transmissions among large nodes and shorter transmissions where one of the nodes is small. The threshold population size distinguishing large and small populations (also fitted) was estimated at approximately 158,000.

**Table 3:** Maximum likelihood estimators for parameters of the assortative model at the county level.

τ_c_	log(P_c_)	α (km)	π	
0.854	5.19	280	1.28	Common
-	γ	γ_2_	d_c_ (km)	
-	17.4	1.21	164	Large-large
-	20.5	2.32	138	otherwise

Relative likelihood, Δ*
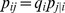
* = −12084243.

**Figure 7:**
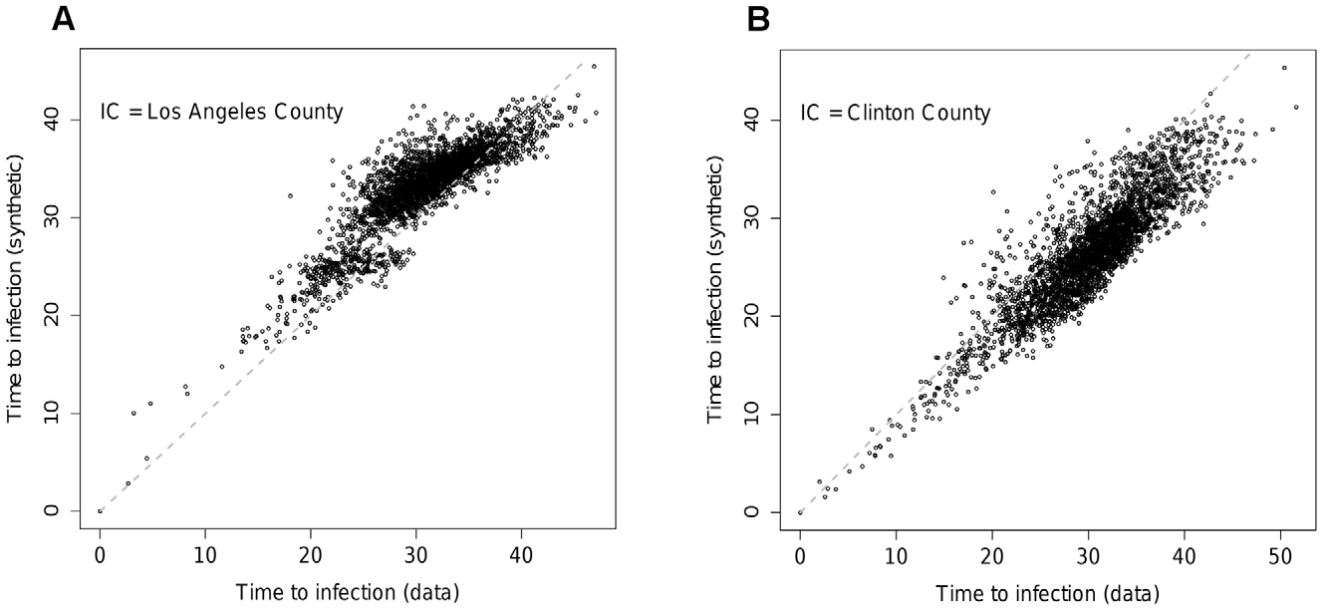
Times to infection for different nodes for the assortative model in the US at the county level of aggregation with initial seeding A) Los Angeles County, B) Clinton County, Iowa. Grey dots represent 95% intervals across simulation realisations for the times to infection on the data network.

The gain in likelihood of the assortative model over the simpler version is not large. However, the improvement in the ability to reproduce the epidemic timing seen for the data network is significant (See Figure 5D for the corresponding 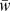 value). The improvement in the quality of the fit in terms of epidemic behaviour can be seen Figure 7. Figure 7A and B should be compared with Figure 5B and C respectively. The assortative structure has clearly lessened some of the bias towards transmission being too fast to smaller nodes and too slow to larger ones in the epidemic initiated in Los Angeles (Figure 7A). Equally, the epidemic started in Clinton County has ‘slowed down’, converging towards the behaviour seen for the data network (Figure 7B). These improvements in fit are reflected in the RMS time differences shown in Figure 5D.

## Discussion

Understanding what aspects of human movement patterns are important to capture in transmission models is important in improving our ability to predict the spatiotemporal spread of emerging epidemics. As increasing volumes of finely resolved mobility data become available, one option is to incorporate these data directly into epidemic simulations. However, the availability of such directly applicable and comprehensive data sets is confined largely to Western Europe and the United States and concerns primarily human movement. In many parts of the world, such as Africa and East Asia, such data may be available only patchily, if at all, or at an inconvenient scale of aggregation [17], [18]. Hence models are necessary to extrapolate to data poor areas. In this paper, we have looked at how well gravity movement models perform when fully supported by data. How well they perform with limited data is a topic for further work.

Optimally, we would like a mechanistic but parsimonious model of human mobility which captures just sufficient detail to adequately represent the spatiotemporal spread of infection. ‘Adequately’ is clearly a subjective term, but a clear minimum criterion is that any model of mobility produces spatiotemporal dynamics that are not qualitatively different from those produced by raw mobility data itself. A much more rigorous criterion might be that a mobility model reproduces the connectivity of specific individual locations sufficiently accurately that the expected time to infection of every location estimated from mobility data is reproduced by a model to a certain level of precision.

In this paper, we have focussed on the first criterion rather than the second – we are interested in matching the marginal statistical properties of spatial epidemics at the level of the ensemble of included locations, rather than the risk profile of each individual location.

Our study shows that quite simple gravity models are able to capture many features of UK commuter flows at a variety of spatial scales. However, for a good fit to the observed patterns, it proved necessary to constrain the models to exactly reproduce the number of commuters resident in each node (*i.e.* total outflow, including self-flow). As shown in Figure 1A, the unconstrained model did quite poorly at matching this feature of the data. This seems surprising in light of the fact that the fraction of workers living in a node is well described as a fraction of the total population (approximately 36% in the UK). However, from equation 1, the probability of a worker living in node *i* is
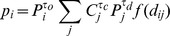
This expression is clearly dependent on the number and ‘attractiveness’ of other nodes around node *i*, indicating that the globally constrained model is density dependent. As result, the model favours greater outflows in more population- and node-dense areas. The locally constrained model largely removes this density dependence.

The introduction of a matched two-part kernel greatly improves the ability of gravity models to reproduce the observed distance distribution of journeys. The improvement to the fit is mainly seen in journeys longer than 200 km which are quite rare in the data and hence give only a modest improvement in likelihood.

The UK data set allows for models to be fitted to three nested levels of aggregation; ward, district and county. As the spatial scale of aggregation decreases, most of the parameters change monotonically (*τ_c_*,*λ* = *α*/*γ*,*γ_2_*,*π*). The theoretical limit of this aggregation process would be at the level of the individual at which point, powers on population sizes are meaningless. Hence the apparent convergence of the parameter *τ_c_* to 1 suggests that gravity models may continue to be valid down to this level. The apparent convergence of π to 1 also suggests the distance kernel at the individual level might be smooth without a discontinuity at 0. This is encouraging for the use of gravity models in individual-based micro-simulations [10], [19].

Epidemics run on synthetic mobility networks derived from the best-fit UK model match quite closely simulated outbreak behaviour on the data network. The only clear bias, which is replicated at all levels of aggregation, is faster rates of spread both to and from the smallest nodes than are seen on the data network. For the poorer fitting models (locally constrained SK and globally constrained MK), their ability to reproduce epidemic dynamics on the data network is not well predicted by their likelihood values. The globally constrained MK performs comparably to the locally constrained model, but the SK model is unable to reproduce the timing of infection of the nodes infected latest in an epidemic (Figure 2C). This is because infection does not spread to distant nodes in a wave-like manner (*i.e.* utilising long chains of strong, short distance connections), but rather through weaker, long distance connections from the first few infected nodes. As a result, biases in the models in the strengths of rare long distance connections can have an effect on an epidemic out of all proportion to the effect of those long-range connections' contribution to the likelihood.

The transmission time theory developed in SI Text, S1, proves to be a good predictor of the times to infection generated by simulation, although with a slight tendency to overestimate times to infection. It represents the sensitivity of epidemic dynamics to the epidemiological parameters well, both qualitatively and quantitatively. The approximation gives a fairly accurate prediction for first infection times at the UK county level (Figure 3C), under the assumption that infection travels between nodes via the minimum-time route only. However, at the district level, this approach starts to break down. While infection times of nodes affected early in an epidemic are well described, later infection times are considerably over-estimated. This appears to be because some nodes are subject to significant forces of infection from more than one source node. Close examination of infection times indicate that transmission through large conurbations is accelerated in comparison with the naive single quickest path algorithm. Neighbouring nodes in cities are very well connected and hence generate many sources of force of infection for a susceptible node. A related issue is that the assumption of small proportional flows between nodes underpinning the analysis may be broken between densely populated contiguous nodes. Essentially, nodes within conurbations are often so strongly connected that they cannot be regarded, epidemiologically, to be independent weakly interacting communities. An equally accurate but more parsimonious division of the population geographically could be achieved by amalgamating such urban nodes. The time to infection expression we derived could be used to discriminate which nodes should be amalgamated by defining a minimum transmission time for two nodes to be considered independent. This is an area for future work.

When applied to the US data, the rank order of fit of the different gravity models examined was the same as for the UK analysis, but the quality of fit was considerably worse for the US county-scale data (the finest level of aggregation considered). The mean deviance measure, 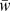, for the best fit MK model makes this clear (Figure 5D). Although the predicted node inflows match the data well, reproduction of the distance distribution of journeys is not particularly accurate (Figure 4).

Comparing epidemics run on the best fit synthetic and data networks for the US shows a much poorer fit than obtained in the UK analysis to times to infection, in line with what might be expected from the mean deviance statistic. The much improved fit at the state level compared with the county level suggests that some of the problem in matching the data arises from heterogeneities in movements and population at the county scale. A number of refinements of the basic MK model were attempted, such as: inclusion of a mean income term and population density term alongside the population terms in equation 2; inclusion of a third matched kernel section to better match the distance distribution (see Figure 4B). None of these produced any appreciable improvement in likelihood or epidemic fit. However, the deviances in time to infection of nodes shown in Figure 6 suggest a limited number of flows into and out of the most populated nodes are underestimated by the model. Addressing this with an assortative spatial interaction between large and small populations improves the fit between epidemics. The principle change in the model is an increase in the distance over which movements can occur between large population centres.

A plausible explanation for the excess in long-range journeys between large nodes in the US is domestic air travel. The extreme size of the continental United States and its sparsely populated central region encourages long range flights to connect the two coasts, for example, usually between highly populated nodes. There is much less need for flights over short distances and few closely spaced large population nodes for them to fly between. These kinds of flows, although important for disease transmission, may not be well modelled by power law kernels and gravity models. As they are relatively rare, they carry little statistical ‘weight’ within the likelihood expression, but still play a crucial role in long distance disease transmission.

Comparison between the parameter estimates for the two regions are hard to make, as the levels of aggregation differ. Perhaps the closest match is between US counties and UK districts, both in terms of population size and demographically (large conurbations are made up of several of the units in both cases). As can be seen from tables 1,2 and 3, parameter values are not generally transferable. The exponent of long distance connections is consistent between MK models (approx 1.8), but it is this aspect of the US fit which changes most with the introduction of assortativity and hence is most strongly associated with the poor performance of the MK model in the US. As discussed, the poor fit in the US is probably the result of the demographic heterogeneity and large size of the country. It seems more likely that parameter estimates for the UK may transfer better to other European countries with similar demographies.

It is clear from Figure 6 that although an assortative model considerably improves the fit to the spatio-temporal dynamics of the epidemic, it doesn't have much effect on the underlying biases of the gravity model with regard to the US mobility data. Further work is necessary to identify what features of the data are essential to an accurate model. Preliminary show that dividing the country into regions of similar population density (east and west coasts separated by a central region) leads to better fits within each but requires additional models for movements between them. A recent paper Simini et al. presents a radical new model defining the relative probabilities of trips purely in terms of resident population distributions [20]. Simulations show that this model performs at least as well as the assortative model described in this paper in matching epidemic behaviour. The time to infection theory (SI Text S1) suggests several applications. In meta-population models, it allows a comparison between local epidemic timescale and that of transmission to neighbouring nodes. Hence it is possible to optimise meta-population structure to ensure that individual nodes represent largely independent populations weakly linked to each other. The theory also suggests an alternative approach to clustering on a network in terms of its accelerating effect on the speed of epidemics on a network.

## Supporting Information

Text S1 **Transmission time theory.**
(PDF)

Text S2 **Interpretation of the likelihood.**
(PDF)
