# Supplementary material for: Evaluating the Adequacy of Gravity Models as a Description of Human Mobility for Epidemic Modelling
Source: PLoS Comput Biol. 2012 Oct 18;8(10):e1002699. doi: 10.1371/journal.pcbi.1002699 (PMC3475681; doi:10.1371/journal.pcbi.1002699)
Supplement: Text S1 — Transmission time theory. (PDF) [file pcbi.1002699.s001.pdf]

## Transmission time theory

In this section, we derive an approximate expression for the mean time taken for infection to transmit from one node to another. Within the simulation, the infection time of a node is taken as the time at which the first resident of that node becomes infected. Because of the structure of the movement network, there are two routes of transmission from node A to node B. Infection can be carried to B from A by a resident of A who works in B (export mode) or infection can be brought back to B by an resident of B working in A. (import mode). The two modes are closely related and we will only discuss the export mode in detail. In fact, transmission dynamics are two-stage in both modes, complicating the analysis. A number of assumptions are necessary to arrive at a simple form for the mean time and the implications of these will be discussed.

Transmission of infection by the export mode involves three populations and two transmission events. We assume that, for any given node, the majority of the population lives and works in the same node. The reliability of this assumption clearly depends level of aggregation of the population. For the data sets considered in this paper, it is sufficiently the case for all levels of aggregation barring the ward level in the UK. A consequence of this assumption is that an epidemic on a node will grow fastest in the population that lives and works in the same place, since these individuals are subject to both the home and work forces of infection (as discussed in the Models section). In the export transmission mode, a localized epidemic among individuals living and working in node A transmits to the population living in A and working in B, from which it transmits to individuals living in B, completing the transmission from A to B. We can write this as AA $\rightarrow$ AB $\rightarrow$ BB, where the two letters refer to the residence and work node of the sub-population. For I0 individuals introduced into population AA at t=0, prevalence at time t is

$$I_{AA}(t) = I_0 e^{rt}$$

where *r* is the growth rate of the epidemic. The force of infection experienced by the population of AB as a whole is therefore

$$\lambda_1(t) = \beta_h \Lambda_1 I_0 e^{rt}$$

where

$$\Lambda_1 = \frac{N_{AB}}{N_A}$$

From our assumption about population movement, $\Lambda \ll 1$. The force of infection generated by individual in AB on the whole of population BB is given by

$$\rho_1 = \beta_w \frac{N_{BB}}{\bar{N}_B} = \beta_w \varsigma_1$$

where $\bar{N}_B$ is the population working in node B. given our assumption about the proportion of the population living and working in the same node, $\varsigma_1 \simeq \beta_w$. We can write down the master equations for the probability that population AB contains *n* infected and population BB is still unaffected.

$$\frac{d}{dt} P_n = \lambda_1(t)(P_{n-1} - P_n) - n\rho_1 P_n - \sigma(nP_n - (n+1)P_{n+1})$$

In this equation, it is assumed that the force of infection experienced by AB is dominated by epidemic in node A. The probability that infection has not yet spread to the destination node is $\sum_n P_n(t)$. In the import mode of transmission, the transmission sequence is AA $\rightarrow$ BA $\rightarrow$ BB. Strictly speaking, this path would register as a transmission when infection reached the BA population since these individuals live in node B. However, we are interested in transmission times within infection chains and hence it is appropriate to consider transmission times between the same kinds of sub-populations. The force of infection on BA is

$$\lambda_2(t) = \beta_w \Lambda_2 I_0 e^{rt}$$

where

$$\Lambda_2 = \frac{N_{BA}}{\bar{N}_A}$$

while that experienced by the population of BB by an infected individual in BA is given by

$$\rho_2 = \beta_h \frac{N_{BB}}{N_B} = \varsigma_h q_2$$

Hence we can construct the master equations for the joint probability that AB and BA contain *n* and *m* infected, respectively, and that BB is uninfected.

$$\frac{d}{dt}P_{n,m} = \lambda_1(t)(P_{n-1.m} - P_{n,m}) + \lambda_2(t)(P_{n,m-1} - P_{n,m}) - (n\rho_1 + m\rho_2)P_{n,m}$$
$$-\sigma((n+m)P_{n,m} - (n+1)P_{n+1,m} - (m+1)P_{n,m+1})$$

The probability that the target population, BB, has not been infected by time *t* is given by

$$\Pi(t) = \sum_{n,m} P_{n,m}(t)$$

By converting the master equations into a PDE for the probability generating function of $P_{n,m}$(t), we find

$$\Pi(t) = \exp\left\{ -\sum_{i=1,2} \frac{\Lambda_i \varsigma_i}{r(\varsigma_i + \sigma + r)}(e^{rt} - 1) - \sum_{i=1,2} \frac{\Lambda_i \varsigma_i}{(\varsigma_i + \sigma + r)(\varsigma_i + \sigma)}(e^{-(\varsigma_i + \sigma)t} - 1) \right\}$$

To simplify this expression, we note that the first term is growing exponentially with respect to the second and will rapidly dominate the expression. Hence we can neglect the second term. Mean time to infection can be calculated from this distribution as

$$\bar{t} = -\int_0^\infty t \frac{d\Pi}{dt} dt$$

Noting that the Λ parameters are small, we can calculate the mean as an approximation for small Λ, giving

$$\bar{t} \approx \frac{-1}{r}\left\{ \ln\left( \frac{\beta_w \beta_h \Lambda_1 \varsigma_1}{\beta_w \varsigma_1 + \sigma + r} + \frac{\beta_w \beta_h \Lambda_2 \varsigma_2}{\beta_h \varsigma_2 + \sigma + r} \right) - \gamma \right\} \tag{1}$$

where γ is the Euler-Mascheroni constant. The growth rate in the source node is approximately given by $r = \beta_w + \beta_w - \sigma = (R_0 - 1)/\sigma$. Initial seedings are placed in the population working in their home node which means that this stage is dominated by the import mode, giving an adjusted expression

$$\bar{t} \approx \frac{-1}{r}\left\{\ln\left(\frac{\beta_w \beta_h \Lambda_1 \varsigma_1}{\beta_w \varsigma_1 + \sigma + r} + \beta_w \Lambda_2\right) - \gamma\right\}$$

The sensitivity to epidemic parameters of network transmission can be gauged from equation 1. For reasonable parameter values, however, sensitivity to epidemic parameters is dominated by the 1/*r* term on the l.h.s. of equation 1. Hence the quantity $r\bar{t}$ is effectively constant. In terms of fractional changes,

$$\frac{\Delta\bar{t}}{\bar{t}} = \frac{1}{1 + \Delta r / r} - 1$$

The expression 1/*r* is the timescale of exponential growth in a given node. Hence the sensitivity of the mean time to infection is a primarily a product of the exponential growth rate rather than the particulars of transmission between nodes. Since 1/*r* is a multiplicative term, all transmission times will be effected proportionally and variation in the epidemiological parameters in a uniform speeding up or slowing down of transmission for all nodes. This effect is demonstrated in the Results section.
