# Supplementary material for: Evaluating the Adequacy of Gravity Models as a Description of Human Mobility for Epidemic Modelling
Source: PLoS Comput Biol. 2012 Oct 18;8(10):e1002699. doi: 10.1371/journal.pcbi.1002699 (PMC3475681; doi:10.1371/journal.pcbi.1002699)
Supplement: Text S2 — Interpretation of the likelihood. (PDF) [file pcbi.1002699.s002.pdf]

Interpretation of likelihood values

By analyzing the expression for the likelihood of the commuter flows predicted by the model, we can get some insight into its interpretation. This allows us to compare the goodness of fit across different data sets and allows us to connect the goodness of fit to the epidemic dynamics on the associated network.

The basic expression for the log likelihood of the movement data for a particular model is given in equation 4. The maximum possible value is given by the saturated model in which journey probabilities match those from the data, $\hat{p}_{ij}$. Hence

$$\hat{p}_{ij} = T_{ij} / T_{Tot}$$

giving a saturated log likelihood

$$\mathcal{L}_{\varnothing} = \sum_{ij} T_{ij} \ln(T_{ij}) - T_{Tot} \ln(T_{Tot})$$

For a particular model, the predicted journey probabilities will differ from the saturated values. For a locally constrained model,

$$p_{ij} = \hat{q}_i (\hat{p}_{j|i} + \delta p_{j|i})$$

where $\hat{q}_i, \hat{p}_{j|i}$ are the relevant saturated values and $\sum_j \delta p_{j|i} = 0$ .The log likelihood for the model is given by

$$L = L_{\varnothing} + \sum_{i,j} T_{ij} \ln\left(1 + \frac{\delta p_{j|i}}{\hat{p}_{j|i}}\right)$$

Assuming that $\hat{p}_{j|i} \gg \delta p_{j|i}$, the difference between the log likelihood of the model and the saturated value can be expanded as

$$\Delta\mathcal{L} \simeq \frac{1}{2} \sum_{ij} T_{ij} \left(\frac{\delta p_{j|i}}{\hat{p}_{j|i}}\right)^2$$

This approximation assumes that the model is sufficiently good that it doesn't predict any strong flows where none are present in the data. We can now define a measure of mean deviance from the saturated model that is independent of the size of the dataset

$$\overline{w} = \sqrt{\frac{1}{T_{Tot}} \sum_{ij} T_{ij} \left(\frac{\delta p_{j|i}}{\hat{p}_{j|i}}\right)^2} = \sqrt{\frac{2\Delta\mathcal{L}}{T_{Tot}}}$$

This quantity is the root of the squared fractional deviation from the saturated model, weighted by the connection strength. It allows us some insight into the quality of fit of models, even across different datasets. In particular, note that quantity $p_{j|i}$ is identical to Λ_1 and closely related to Λ_2 in the expression for time to infection (the numbers of workers in node is closely related to the number of residents).

Considering only the export mode of transmission, the time to infection expression (main text:equation 5) can be broken into a sum of elements including $\ln(\Lambda_1)/r$ covering the impact of the residents of the source working in the target node. Treating $\overline{w}$ as the average fractional error in Λ_1, the average error in $\ln(\Lambda_1)/r$ is $\ln(1+\overline{w})/r$. Hence we might expect the quantity $\overline{w}$ to have at least a strong correlation with the fit for first infection times between data and synthetic networks.
